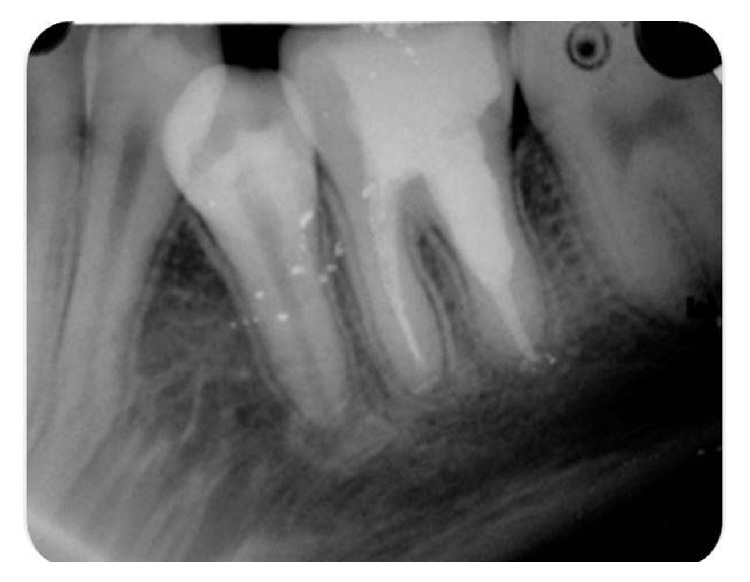# Management of a Large Internal Resorption Lesion with Metal Reinforced Glass Ionomer Cement

**DOI:** 10.1155/2014/205028

**Published:** 2014-11-04

**Authors:** Gurdeep Singh Gill, Atool Chandra Bhuyan, Suraj Arora, Kunal Sethi, Rajwinder Kaur, Tarun Kalra

**Affiliations:** ^1^Department of Conservative Dentistry & Endodontics, JCD Dental College, Jan Nayak Ch. Devi Lal Vidyapeeth, Sirsa, Haryana 125201, India; ^2^Department of Conservative Dentistry & Endodontics, Regional Dental College, Guwahati, Assam 781032, India; ^3^Department of Pedodontics & Preventive Dentistry, JCD Dental College, Jan Nayak Ch. Devi Lal Vidyapeeth, Sirsa, Haryana 125201, India

## Abstract

Mineral trioxide aggregate is the mainstay of treatment of large internal resorption defects. But its cost may be a deterrent to its use in some patients. The present case report describes the successful endodontic management of an extensive internal resorptive lesion in a mandibular molar with metal reinforced glass ionomer cement.

## 1. Introduction

Root resorption is the loss of hard dental tissue (i.e., cementum and dentine) as a result of odontoclastic cell action [1]. It is broadly classified into internal and external resorption [2]. Internal root resorption is rare in permanent teeth and is characterised by oval shaped enlargement of the root canal space [3].

True cause of internal resorption is unknown but it is generally accepted that damage to the organic sheath, predentin, and odontoblast cells covering mineralized dentine inside the root canal exposes the mineralized tissue to pulpal cells with resorbing potential [4]. Trauma (most frequently), pulpitis, and restorative procedures have been suggested as predisposing factors responsible for the damage to predentin.

If not treated timely it can cause extensive damage to tooth structure leading to root perforation and eventually to loss of tooth [2]. Mineral trioxide aggregate (MTA) has become the mainstay in the treatment of internal resorption owing to its superior sealing properties but its high cost, especially in developing countries, can be a deterrent to its use.

The following case report describes the successful management of a large internal resorption lesion in mandibular molar with metal reinforced glass ionomer cement.

## 2. Case Report

**Figure 1:**
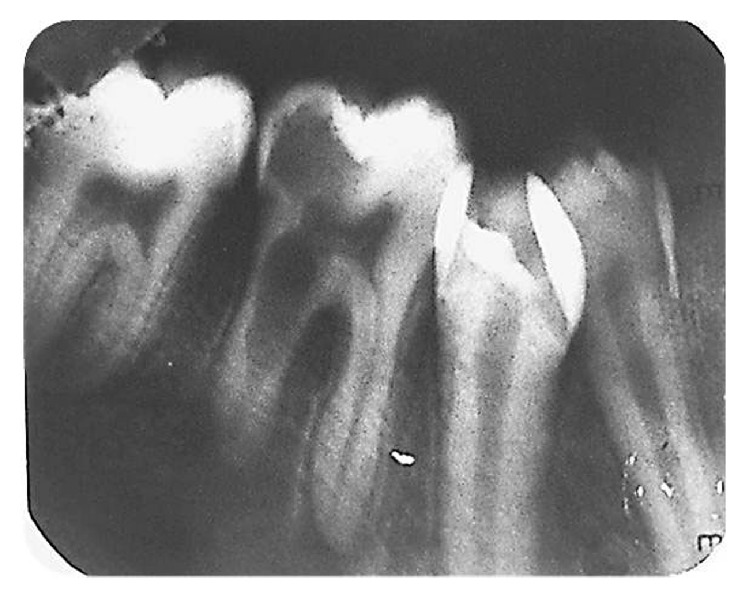


A 12-year-old patient reported to the Department of Conservative Dentistry and Endodontics with complaint of pain and swelling in the mandibular right first molar (tooth number 46, FDI nomenclature) since 2-3 weeks. Pain was mild and intermittent and was relieved by medications. There was no relevant dental or medical history. On clinical examination, tooth had a deep carious lesion and was tender on percussion. Mucosa overlying the roots of 46 was tender on palpation and intraoral swelling was present. Radiographic examination revealed an asymmetrical radiolucency involving coronal and middle part of the distal root as shown in Figure 1. Outline of root canal was not visible in that area. Periodontal ligament was widened and periapical radiolucency was present in both roots.

Based on clinical and radiographic findings, a diagnosis of internal inflammatory resorption with apical periodontitis with respect to (w.r.t.) 46 was made.

Treatment plan involving root canal treatment and sealing the defect with MTA was told to the patient's parents. The parents of patient were unable to afford the treatment cost. In order to save the tooth, an alternative treatment plan involving use of Miracle Mix GIC was implemented after consent of parents.

**Figure 2:**
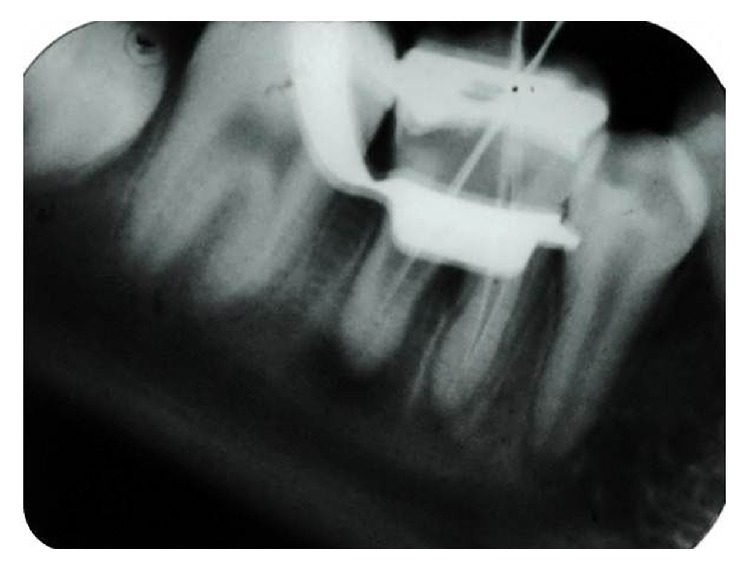


Tooth was isolated and access opening was done under local anaesthesia. There was profuse bleeding from the distal canal. Bleeding was controlled by using thorough irrigation with 2.5% sodium hypochlorite (Asian Acrylates, Mumbai). Number 80 K-file (Mani Dental, Japan) and a sharp curette were used to remove the granulation tissue from the internal resorptive defect area in distal canal. Working length was determined (Figure 2) and pulp was removed from the apical area of distal canal and mesial canal. Calcium hydroxide paste (Vitapex, J. Morita Corp.) was placed in both root canals and patient was recalled after 1 week.

On second appointment, instrumentation was completed with hand ProTaper Universal System (Dentsply Tulsa Dental, OK) in mesial canal and apical third of distal canal. In resorptive area, number 140 K-file was used to clean the remaining area. Calcium hydroxide dressing was done again for a week.

On third appointment, after drying the canals, mesial canals were obturated with ProTaper F2 gutta percha cones. Apical third of the distal canal was sealed with ProTaper F2 gp point and remaining point was seared off with hot plugger just below the lesion.

**Figure 3:**
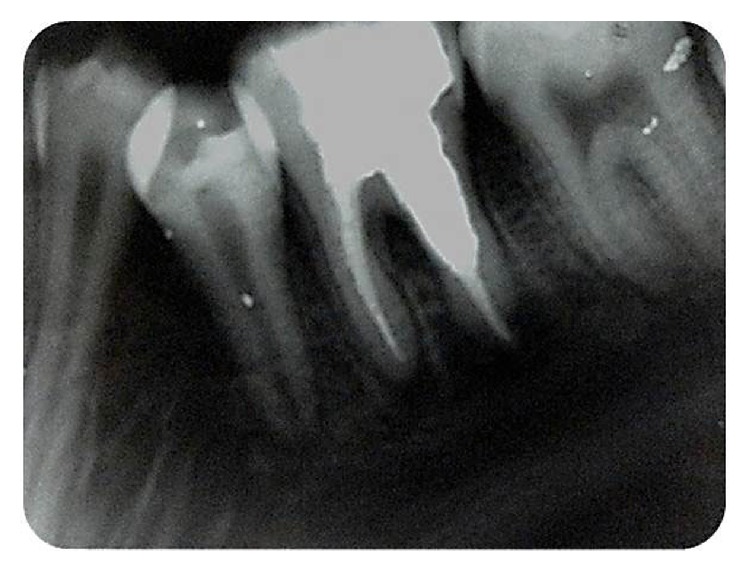


The resorptive defect was filled with Miracle Mix GIC (GC Corp, Tokyo, Japan), using pluggers and condensers with light forces. Access cavity was also filled with Miracle Mix GIC (Figure 3).

Patient was recalled for regular check-ups. After one year (Figure 4), patient was asymptomatic and radiographic healing in the periapical area was almost complete.

## 3. Discussion

Management of a lesion starts with diagnosis. In the current case, diagnosis was clear from the radiograph as root canal outline was not visible in the resorptive defect area. Unlike most cases, patient was symptomatic as lesion was in advanced stage. In internal resorption cases, brisk bleeding from the canal and the distorted shape of canal make instrumentation and location of canals difficult. Weakened tooth structure is another consideration. To control the bleeding, majority of pulp was removed in first appointment. Calcium hydroxide paste placed in root canal further helped in reducing bleeding by necrotizing the remaining pulp tissue and making it more soluble to sodium hypochlorite.

To locate the canal in apical third of distal canal, K-files were precurved 2–4 mm from the tip to avoid any perforation. Once glide path was prepared, hand ProTapers (not rotary) were used for better tactile sensation and ease of obturation (compared with K-files) to avoid exerting any lateral condensation forces on the already compromised roots.

Calcium hydroxide decreases the bacterial load, arrests osteoclastic activity, and stimulates the repair. High pH of calcium hydroxide neutralises lactic acid secreted by macrophages and osteoclasts and inhibits collagenases [5].

Different root canal filling materials have been used in the literature for permanent filling of the root canal in internal resorption cases. Warm gutta percha has been generally preferred in older case reports [6–9]. MTA has been preferred in modern literature [10–13] because of its better seal and biocompatibility [14]. Its high cost was deterrent to its use in the present case.

Composite materials have also been suggested in teeth with a large resorption cavity in the coronal third of the root canal in order to strengthen the tooth and make it more fracture resistant [9]. As the resorptive defect extended deep into the root, there were concerns about the bonding and application of composite in the defect.

In the present case, patient could not afford the cost of MTA treatment and extraction of a crucial tooth at such young age would have been very traumatic for the patient and detrimental for oral health. So, the authors decided to use Miracle Mix glass ionomer cement in the case. Glass ionomer cements have been used successfully in the management of internal resorption cases in the past [15].

Miracle Mix GIC is easily available and economical (compared to MTA), has high strength, bonds to tooth structure, and can be easily introduced into a large cavity without requirement of additional or costly equipment. It is mainly used as a core build-up material; extending the material into the resorption cavity in the present case will further help in retention of core and final restoration of tooth with a crown.

## 4. Conclusion

Even teeth with large resorptive lesions can be saved with timely endodontic intervention. To save a tooth, patient should always be offered alternative choices if he/she is unable to afford the best option. Metal reinforced GIC can be used in managing large internal resorptive lesions.

## Figures and Tables

**Figure 4:**